# Why Test Purchased Cattle in BVDV Control Programs?

**DOI:** 10.3389/fvets.2021.686257

**Published:** 2021-08-27

**Authors:** Linda Van Duijn, Inge Santman-Berends, Marit Biesheuvel, Jet Mars, Frederik Waldeck, Gerdien van Schaik

**Affiliations:** ^1^Royal GD, Deventer, Netherlands; ^2^Department of Production Animal Health, Faculty of Veterinary Medicine, University of Calgary, Calgary, AB, Canada; ^3^Department of Population Health Sciences, Faculty of Veterinary Medicine, Utrecht University, Utrecht, Netherlands

**Keywords:** BVD, eradication, PI, bovine viral diarrhea, control program, Trojan cow, BVDV

## Abstract

Bovine viral diarrhea (BVD) is controlled in many countries by detection and culling of persistently infected (PI) animals. The most important risk factor for BVDV introduction is purchase. An introduced cow can be PI and transmit the virus to other cattle in the herd. If she is not PI but is pregnant, there is still a risk because the subsequently born calf may be PI, when she encountered the virus in early pregnancy. To control this risk, all cows > 1 year from non-BVDV-free herds that are introduced in herds that participate in the Dutch BVDV control program are tested for virus and antibodies. Depending on the results, subsequent measures such as suspension of the BVDV-free status, removing the animals from the herd, or testing the off-spring of the cow for virus, are undertaken. The aim of this study was to evaluate the results of this risk mitigating measure. Data on cattle movements, calving's, herd-level BVDV status, and animal-level test data were available from all dairy herds that participated in the national BVDV control program (>14,000 dairy herds) for the year 2019. The data were combined and parameters of interest were calculated, i.e., (i) the number of purchased BVD virus positive cattle and (ii) the number of BVD virus positive calves born from purchased cows within 9 months after introduction. In 2019, 217,301 cattle were introduced in Dutch dairy herds that participated in the BVDV control program. Of these, 49,820 were tested for presence of BVD virus and 27 (0.05%) cows introduced in 21 different herds tested BVD virus positive. Out of 46,727 cattle that were tested for antibodies, 20.5% tested positive. The seropositive cows produced 4,341 viable calves, of which 3,062 were tested for virus and subsequently, 40 (1.3%) were found BVD virus positive. These 40 BVD virus positive calves were born in 23 herds. The risk mitigating measure led to detection of 67 BVD virus positive animals in 44 unique herds in 2019. This study makes plausible that the probability and impact of re-introduction of BVDV can be minimized by testing introduced cattle and their subsequently born calves.

## Introduction

Bovine viral diarrhea virus (BVDV) is a pestivirus belonging to the *Flaviviridae* family (1). It was first discovered in New York dairy herds in 1946 and in the same year in Canada and is since an endemic cattle disease in many parts of the world (2). An important feature of the epidemiology of BVDV is the existence of persistently infected animals (PIs). When a pregnant animal encounters the virus for the first time between day 42 and 125 of gestation, the immune system of the fetus is not fully developed and therefore the virus will persistently infect the fetus (3, 4). These cattle pregnant with PIs are called Trojan cows. At the same time the pregnant animal will develop antibodies against the virus. Due to the continuous shedding of large amounts of virus, PIs are the most important source of the virus and the main reason why herds remain infected (5). Besides this vertical route of infection, BVDV can also spread horizontally. When this occurs the transiently infected (TI) animal will start an immune response and clear the virus. This way, although limited, also contributes to the spread of the virus (6). The disease causes economic losses for the cattle industry (7, 8) and has detrimental effects on animal welfare. In prevalence studies in different European countries, as well as the Netherlands, BVDV was found to be present (2, 9). Therefore, several European countries or regions have implemented bovine viral diarrhea virus programs (10) to control and eradicate the virus.

These control programs aim to detect and remove PIs, and results over time give insight into their success (11). When eradicating BVDV from a country or region, it is important to know the risk factors for (re)introduction of the virus. Therefore, many risk factor studies have been carried out over the years, including a meta-analysis by Van Roon et al., (12) in which frequently found risk factors, e.g., herd type, herd size, participation in shows or markets, introduction of cattle, grazing, and contact with other cattle herds on pasture were quantified. In this meta-analysis, introducing cattle into a herd appeared a significant risk factor for having a BVDV infection. Furthermore, the purchase of pregnant heifers is associated with a higher risk of introduction of BVDV infection into a herd (13). Earlier studies investigated to which degree movement restrictions of female animals, over 12 months of age, from infected herds, would prevent Trojan births in other herds (14). However, the proportion of introduced female cattle over 12 months of age, that give birth to a PI is unknown.

In the Netherlands, about 50 percent of dairy herds regularly purchase cattle (15). These cattle are mostly purchased from other dairy herds, often with support of a trader. Purchase patterns are equally distributed across the year. Trading of breeding cattle through cattle markets or collection centers is not allowed. Cattle moved from one herd to another herd may be transported with cattle from other herds. PI or TI cattle can thus infect naïve cows during transport and these may subsequently result in the risk of introducing BVDV in herds with a BVDV-free status, either through purchase of a PI or through purchase of a trojan cow. Therefore, besides testing for presence of virus, all female cattle over 1 year of age, originating from a non-BVDV-free herd, that are introduced into a dairy herd that participates in the BVDV control program, are tested for BVDV antibodies. If a cow tested antibody positive, the new-born calf (Trojan calf) needs to be tested for BVD virus, even though antibodies found could also be the result of vaccination. About 20% of dairy herds vaccinate for BVDV in the Netherlands. The aim of this study was to evaluate the effectiveness of this risk mitigating control measure. Other countries embarking on a national program or countries searching for risk-mitigating improvements for their current BVDV program may also benefit from these results.

## Materials and Methods

### BVDV Program

In the Netherlands, a voluntary BVDV control program has been in place since 1997. In this control program, cattle herds can obtain a BVDV-free status after a full herd screening for BVD virus (16). In 2018, participation in the control program became mandatory for dairy herds. The original program was slightly changed, and three alternative routes to become BVDV-free were introduced: prolonged evaluation of antibodies in young stock serum samples, regular bulk milk screening and ear notch sampling. For non-dairy herds, participation remained voluntary. The Dutch cattle industry is committed to eradicating BVDV, and the Dutch government is expected to require mandatory participation by all Dutch cattle herd in one of the BVDV programs in due time. For more details on the original BVDV-free program see the full program description in (9). The different routes of the Dutch BVDV program are described in more detail in (17).

### Testing Introduced Cattle

Testing of introduced cattle is mandatory in all routes and independent of the BVDV status of the herd of destination. When cattle, unless from BVDV-free herds, are introduced into a herd, the herd's status will be suspended and only regained when the introduced cattle have a negative test result for BVD virus. Besides, cows older than 1 year need to be tested for the presence of antibodies. Cows that test positive for antibodies will cause further suspension of the BVDV-free status, even when they are BVD virus negative. When either (i) the cow produces a calf within 9 months after purchase that tests negative for BVD virus, (ii) if no calf is born within 9 months after introduction, or (iii) the cow is removed before calving, the herd will regain the BVDV-free status.

### Diagnostic Testing

Within the BVDV programs, test results are accepted from laboratories that have their BVD test accredited by Wageningen Bioveterinary Research (WBVR), the Dutch national reference institute.

Diagnostic testing in the BVDV program is being supervised by the Dutch national reference institute (WBVR, Lelystad) and all tests must meet specific requirements. Tests for virus must have a sensitivity of at least 99.5% and specificity of 99%. Antibody tests must have a diagnostic sensitivity and specificity of 98% or higher.

### Available Data

To evaluate the risk of purchase, all data on purchased cattle and their subsequently born calves were assessed for 2019. Four datasets were available that provided census data on all cattle located on Dutch dairy herds that participated in the national BVDV program (>14,000 herds in 2019):

- Cattle movement data that is registered in the national identification and registration (I&R) database (Netherlands Enterprise Agency, Assen the Netherlands). These data contain movement level data with the unique herd number (UHI) of the herd of origin, the destination UHI, the unique animal ID, the date of movement, the reason of movement.
- Calving data, registered in the I&R database: these data contain the unique animal ID of dam and calf and the calving date.
- Herd-level data of the national BVDV program (Royal GD, Deventer the Netherlands). These data contain the UHI, the chosen BVDV-free route, the status within that route (e.g., infected, under control, suspended, unsuspected or free) with the start and, if present, the end date.
- Animal-level test data (ZuivelNL, the Hague the Netherlands) with the unique animal ID, the type of test (virus or antibodies), the matrix (tissue, serum or milk), the sampling date, the date the result was available, the test result.

### Validation and Analysis

The data-validation and analyses were conducted in seven serial steps. First, the movement data were combined with the data of the BVDV control program and only movements of cattle introductions in dairy herds that participated in the BVDV control program were retained. Subsequently, the cattle were stratified into two groups indicating whether the introduction involved intracommunity cattle movements or intercommunity cattle movements. The movement data were combined with the BVD test results on the level of the animal and the date of introduction. Data from cattle without test results were removed from the data. These included (i) cattle originating from countries assumed BVDV-free in 2019 (i.e., Denmark), (ii) cattle originating from herds that were classified as BVDV-free, (iii) cattle < 1 year old with an available virus result and (iv) cattle originating from a veterinary entity (VE). In the Netherlands, VEs are defined as dairy herds having one or two holdings in different locations with regular exchange of cattle (>5 times/year). Generally, in one of these locations, the young stock is housed. The BVDV status of these locations are considered the same. Therefore, in the BVDV program, cattle movements between locations of a VE will not require testing. In the fourth step the tested cattle were stratified in two groups according to their age: cattle < 1 year old that only needed to be tested for presence of BVD virus and cattle ≥ 1 year old that had to be tested for presence of antibodies and (if not already available) for virus. This resulted in our first outcome of interest: the number of introduced cattle that appear a PI. These BVD virus positive cattle are removed from the destination herd shortly after detection.

The data from cattle that tested antibody positive were subsequently kept for further evaluation. These data were combined with the calving data and the information of the first calving date (including the exact date and the calf ID) were retained. Data from seropositive cows of which no calving was registered within 9 months after the introduction were excluded from the analyses. The data of the cows with a calving date were divided into cows with a calving that did not result in a live born calf (no calf ID is available) and calving's that resulted in live born calves. The data of the live born calves were singled out and combined with the individual testing data. This resulted in our second result of interest: the number of BVD virus positive calves born from newly introduced seropositive dams. Additionally, we evaluated what happened to the calves that were not tested.

## Results

**Figure 1:**
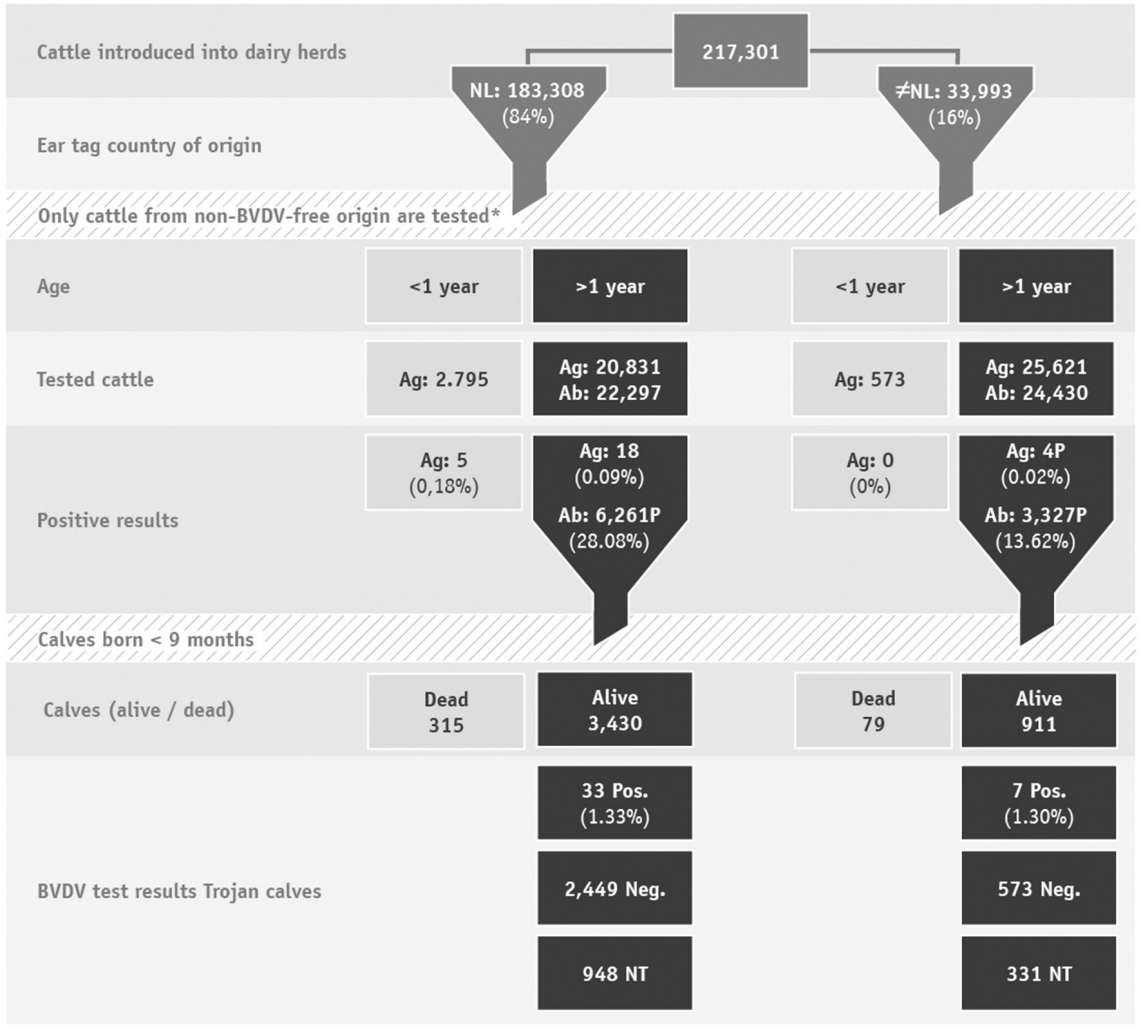
Flowchart of cattle introduced into dairy herds and their test results. *Most introduced cattle originated from a BVDV-free herd, VE or a BVDV-free country, therefore testing is not required. Ag, antigen; Ab, antibody; NT, not tested.

In 2019, Dutch dairy farms introduced 217,301 cattle into their herds, of which 97% were female. These cattle originated mainly from within the Netherlands (84%). The remaining 16 percent originated from outside the Netherlands, of which 80% had German ear tags (Figure 1).

The 217,301 cattle were introduced in 9,331 Dutch dairy herds, which comprises 52% of all Dutch dairy herds. The median number of cattle introduced per herd with introduction of cattle in 2019 was 13. On average herds in which cattle were introduced were larger with a median number of 98 cows (>2 years old) compared to herds without cattle introductions (median 83 cows > 2 years). More descriptive movement information for both herds with and without cattle introductions in 2019 can be found in Table 1. The dairy herds in the Netherlands are distributed across the country with the highest herd density in the Eastern and Northern regions (17).

**Table 1:** Descriptive statistics of all Dutch dairy herds stratified to whether or not cattle were introduced in 2019.

**Herd characteristics**	**Herds with introduction of cattle: median, 25-75 percentile n = 9,331**	**Herds without introduction of cattle: median, 25-75 percentile n = 8,941**
**Herd size**		
- Heads of cattle > 2 years old	98, 68-136	83, 60-114
- Number of calves <1 year old	30, 18-46	29, 20-41
**Influx**		
- Number of births	87, 58-124	57, 53-105
- Number of introduced cattle	13, 4-33	0
**Outflux**		
- Percentage calves moved to veal industry	65%	60%
- Number of cattle (>1 year) moved to slaughter	4, 2-8	3, 2-6
- Number of cattle (>1 year) moved to other herds	0, 0-2	0, 0-0
- Number of deceased cattle (>1 year)	1, 0-2	0, 0-1

Through antibody and virus testing of introduced cattle in 2019, in total 67 BVD virus positive cattle were found in 44 unique herds. From the 217,301 introduced cattle, virus results were available for 49,820 and antibody results were available for 46,727 cattle > 1 year old. For the remaining cows, no BVD diagnostics were required after the introduction because they originated from a BVDV-free herd, a VE, were previously tested for virus or originated from a BVDV-free country (Figure 1).

### Virus Positive Introduced Cattle

Of the 49,820 introduced cattle of which a virus result was available 27 (0.05%) tested BVD virus positive. Of these, 23 originated from the Netherlands, and four had foreign ear tags. The 27 BVD virus positive animals were introduced into 21 dairy herds.

### Antibody Positive Introduced Cattle

A BVDV antibody test result was available for 46,727 cattle, of which BVDV antibodies were detected in 9,588 (20.5%) cattle. Cattle originating from the Netherlands tested antibody-positive more often than cattle originating from another country (mainly Germany), respectively, 28.1 and 13.6%. Of the BVDV antibody-positive animals, 4,527 (47.2%) produced a calf within 9 months after introduction. The 4,527 cows produced 4,737 calves, of which 4,341 were viable (91.6%). The remaining 393 calves were stillborn or died before being ear tagged (Figure 1).

### Trojan Calves

Of the 4,341 ear tagged calves, 3,062 (70.5%) were tested for BVD virus. Forty of the tested calves were BVD virus positive (1.3%). These calves were born in 23 unique dairy herds.

Of the Trojan calves that were not tested for BVD virus (*n* = 1,279), 976 were moved off the farm, the majority of these went to veal calf farms. Given that no result is available for these calves the BVDV status of these dairy herds is suspended for a period of 10 months and during this period all newborn calves have to be tested. Another 143 calves were still present in the herd and these farmers will have re-started in a route to become BVDV-free. Of the untested calves, 158 died, resulting in a mortality risk of 12.4% [95% confidence interval (CI) 10.7-14.4%]. Of the 3,062 calves that were tested significantly fewer calves died (proportion test *P* < 0.0001) compared to the untested group. In total 217 of the tested calves died, resulting in a mortality risk of 7.1% (95% CI: 6.2-8.1%) (Table 2).

**Table 2:** Mortality risk of ear tagged Trojan calves born from antibody positive dams introduced into dairy herds.

**Trojan calves (total)**	**Alive (*n*)**	**Dead (*n*)**	**Mortality risk (%) and 95% confidence interval**
tested (*n* = 3,062)	2,845	217	7.1, 6.2-8.1
not tested (*n* = 1,279)	1,121	158	12.4, 10.7-14.4

## Discussion

The BVDV national herd-level prevalence in dairy herds in the Netherlands declined from 26% (2004) to 8.7% (2016) and the number of BVDV-free and BVDV-unsuspected herds increased (17). Therefore the number of BVD virus positive cattle that were detected by screening of introduced dams and their calves were relevant for the progress toward eradication of BVD on a national level.

Additionally, the number of BVD virus positive calves detected may be an underestimation because not every calf born out of a Trojan cow was tested. The mortality risk in this untested group was higher than the mortality risk among the tested calves. It may be that part of the untested calves died because of a BVD virus infection. Furthermore, mortality risks in calves have been found to be higher in BVDV infected herds (18). Also in herds with identified PI cattle a three-fold rise in calf mortality was seen (19). Nevertheless, given that the presumably BVD virus positive calf died and cannot transmit the virus to other cattle and the fact that the free status of the herd is suspended anyway as a result of incomplete evidence of freedom, the fact that some BVD virus positive animals may have remained undetected has limited impact on BVD virus transmission.

In 2019, testing introduced cattle for BVD virus in the Netherlands led to the detection of in total 67 BVD virus positive animals in 44 herds. Out of these 67 positive cattle, 27 tested BVD virus positive right after the introduction, and 40 additional BVD virus positive calves were detected by screening of newborn calves born out of dams that tested antibody positive at the moment of introduction. This risk-mitigating control measure did not prevent the introduction of BVDV because the BVD virus positive cattle were already added to the herd or the BVD virus positive calf was born in the herd. However, the testing procedure for introduced cattle did result in early detection of BVDV in these herds. By early detection of BVD virus positive cattle, further spread in the farm can be prevented and actions can be taken to regain the free status as soon as possible. The BVDV-free status of these herds is suspended from the moment of introduction of the animal. Unless they prove to be virus negative (cattle < 1 year old) or both virus and antibody negative (cattle ≥ 1 year old), the herd status will remain suspended until 9 months have passed without the birth of a calf or the subsequently born calf is tested BVD virus negative. The implication of the suspended status is that the herd cannot longer trade cattle with a BVDV-free status. This will prevent further spread of the virus by cattle trade with other herds, that are often seronegative due to decreasing BVDV prevalence in the Netherlands (17).

Early detection of introduced BVD virus positive cattle or of BVD virus positive calves is important because the Netherlands has a high cattle density. In a study by Veldhuis et al., (20), the odds of a reintroduction of BVDV increased with the number of non-BVDV-free neighboring herds, herd size and purchase of pregnant cows. Graham et al., (21) also found BVDV infected neighboring farms to be a risk factor for a BVDV infection in Irish herds. Therefore early detection of these non-BVDV-free herds and subsequent actions to rapidly eliminate the infections are important measures to prevent transmission to neighboring herds.

In Ireland the retention of PI calves is a risk for the progress of the control program (22). In the Netherlands, because of the relative low economic value of dairy calves, the awareness of farmers that PI calves can lead to economic losses and the pressure of the dairy cooperation to maintain a BVDV-free status, PI calves are generally not retained. However, we do observe other herd owner behavior that is not beneficial for the progress of the BVDV control program i.e. Trojan calves that were not tested for virus. The farmers seem to lack awareness of the risk of that calf being virus positive, especially when the dam was vaccinated for BVDV. For the individual herd this risk might be negligible but for the progress of BVDV eradication in the Netherlands it is important that these calves are also tested. Why herd owners demonstrate such behavior is complex and warrants a sociological approach. Biesheuvel et al., (23) reviewed international studies on farmer behavior regarding cattle disease control and found that many factors influence farmer behavior. To get a better understanding of farmers' motivators, and to ultimately change their unwanted behavior, it would be beneficial to identify why these farmers do not comply with the program's rules purposely. They concluded that the area on how to change farmer behavior is very complicated. In the Dutch BVDV control program from March 2020 on additional measures were taken to prevent retention of these possible PIs for a prolonged period. The only permitted restart for herds that did not test those calves was in the BVDV-free route with whole herd screening. This costly procedure prevents undetected PIs to remain in the herd for a longer period of time as well as motivates farmers to test the calves of introduced seropositive dams.

Within 9 months of introduction into the dairy herd, about half of the purchased cattle had not produced a calf. This proportion was higher in cattle that originated from other countries then the Netherlands (72.6%). In the Dutch program, all female cattle > 1 year of age have to be tested for antibodies, regardless whether the cow is pregnant or not. Other countries with a BVDV program in place, e.g., Ireland focus on detecting and eliminating of the virus (24) or do not assign herd statuses but instead install movement restrictions for pregnant cattle [e.g., Switzerland, (25)]. Given that only pregnant seropositive cows can be Trojan cows and thus produce a PI, a pregnancy check, could reduce the length of the period with a suspended status for the herds that introduce non-pregnant cows. At this moment, pregnancy checks are not considered within the Dutch BVDV program, but the results of this study warrant further investigation on the costs and benefits of allowing pregnancy checks to reduce the number of cattle that need to be tested for BVDV antibodies and the duration of suspension of the BVDV-free herd status that is currently 10 months.

## Conclusion

The risk of (re)introducing BVDV through purchase of cattle in herds that participate in the national Dutch BVDV-free program is limited. However, the (re)introduction of the virus can have a large impact and result in major economic losses for the individual herd. In a country or region that has a successful BVDV control program in place, the prevalence of BVDV will decrease, which leads to an increased proportion of susceptible cattle in the population. In such situation, the impact of a new outbreak and thus the importance of controlling the risk of purchase increases. Therefore, to support eradication of BVDV in the Netherlands, it remains important to limit the spread of new BVDV infections through introduction of cattle. The virus and antibody testing of purchased cattle has likely been beneficial in preventing spread of infection. This conclusion may also be true for other countries with a BVDV control program in place.

## Data Availability Statement

The raw data supporting the conclusions of this article will be made available by the authors, without undue reservation.

## Author Contributions

LV was the primary author of the manuscript. IS-B conducted the analyses, helped in writing the paper, and proof read the paper. IS-B, JM, FW, MB, and GS edited draft versions of the paper and provided advice for improvement. GS acted as supervisor of the group and provided scientific and epidemiologic input. All authors participated in the writing of this manuscript, have proofread the final version of the manuscript and agree with its contents.

## Conflict of Interest

LV, IS-B, JM, FW, and GS are employed by Royal GD, which runs the national BVDV control program. The remaining author declares that the research was conducted in the absence of any commercial or financial relationships that could be construed as a potential conflict of interest.

## Publisher's Note

All claims expressed in this article are solely those of the authors and do not necessarily represent those of their affiliated organizations, or those of the publisher, the editors and the reviewers. Any product that may be evaluated in this article, or claim that may be made by its manufacturer, is not guaranteed or endorsed by the publisher.
